# Predictive Ability of Waist Circumference and Waist-to-Height Ratio for Cardiometabolic Risk Screening among Spanish Children

**DOI:** 10.3390/nu12020415

**Published:** 2020-02-05

**Authors:** Paola Arellano-Ruiz, Antonio García-Hermoso, Jorge C. García-Prieto, Mairena Sánchez-López, Vicente Martínez Vizcaíno, Montserrat Solera-Martínez

**Affiliations:** 1 Centro de estudios Socio-Sanitarios. Universidad de Castilla La Mancha, 16071 Cuenca, Spainjorge.canete@uclm.es (J.C.G.-P.); Mairena.Sanchez@uclm.es (M.S.-L.); Vicente.Martinez@uclm.es (V.M.V.); Montserrat.Solera@uclm.es (M.S.-M.); 2 Navarrabiomed, Complejo Hospitalario de Navarra (CHN), Universidad Pública de Navarra (UPNA), IdiSNA, Pamplona, 31008 Navarra, Spain; 3 Laboratorio de Ciencias de la Actividad Física, el Deporte y la Salud, Universidad de Santiago de Chile, USACH, Santiago 71783-5, Chile; 4 Facultad de Educación. Universidad de Castilla La Mancha, 13701 Cuidad Real, Spain; 5 Facultad de Ciencias de la Salud. Universidad Autónoma de Chile, Talca 1670, Chile; 6 Facultad de Enfermería, Universidad de Castilla La Mancha, 16071 Cuenca, Spain

**Keywords:** obesity, metabolic risk factors, anthropometric indicators

## Abstract

An excess of fat mass has been associated with adverse cardiometabolic risk factors. Different anthropometric measures have been proposed as alternative non-invasive measures for obesity-related cardiometabolic risk. To evaluate the magnitude of association between waist circumference (WC) and waist-to-height ratio (WtHR) with cardiometabolic risk factors and metabolic syndrome and to determine the WtHR cutoff associated with a more favorable cardiometabolic risk profile in Spanish children, data were taken from a cross-sectional survey conducted in 2010 among 848 schoolchildren aged 8–11 years from 20 public schools in the province of Cuenca (Spain). Anthropometric variables, glucose, insulin, triglycerides (TG), high-density lipoprotein cholesterol (HDL-C), systolic (SBP) and diastolic blood pressure (DBP) and metabolic syndrome (MetS) were also analyzed. WtHR and WC had a good accuracy for TG, insulin, and MetS. The diagnostic odds ratio ranged from 2.95 to 9.07 for WtHR and from 5.30 to 27.40 for WC. The main result of the present study suggests that both WtHR and WC could be used as a screening tool to identify children with cardiometabolic abnormalities.

## 1. Background

The prevalence of childhood overweight and obesity has been growing worldwide during the last three decades [1,2,3]. Obesity and overweight are characterized by an excessive accumulation of body fat, and are associated with adverse cardiometabolic factors (hypertension, dyslipidemia, or insulin resistance) [4]. The presence of these risk factors in childhood is common [5] and clinically relevant because it has short- and long-term physical and psychological consequences, such as depression and an increased risk of type 2 diabetes and cardiovascular diseases in adulthood [6]. These cardiometabolic risk factors can appear independently or cluster together. This clustering is called metabolic syndrome (MetS) and its ethology is not completely understood, thus there is a lack of consensus about the definition in the pediatric population [7].

Different anthropometric measures have been proposed as alternative non-invasive measures for obesity-related cardiometabolic risk. Body mass index (BMI) is frequently used as a surrogate measure of adiposity in children. However, it remains unclear whether the clinical practitioner should assess BMI or WC as a surrogate measure of adiposity [8]. The International Obesity Task Force [2], the U.S. Centers for Disease Control and Prevention [9], and The World Health Organization (WHO) propose the use of BMI for age and gender, using the percentile based criteria, as measure of adiposity and screening tool for cardiometabolic risk [10]; however, BMI by definition cannot distinguish between fat and fat-free mass [11]. Therefore, an elevated BMI might not necessarily reflect increased adiposity [12]. The International Diabetes Federation (IDF) proposes to use the 90th percentile of waist circumference (WC) as the cutoff for defining central obesity [13], due to the fact that WC has been suggested as a good marker for visceral fat [14,15], it seems unlikely that WC and BMI provide an accuracy quantification of visceral fat. However, the use of direct measures of adiposity as percentage body fat does not have any advantage over BMI or WC as surrogate measure of adiposity [16]. Waist circumference (WC) has been used as an indicator of central adiposity with high sensitivity and specificity. This indicator has been presenting more accurate positive associations with cardiovascular risk factors than the BMI [17]. The use of the 90th percentile of WC as a threshold for defining abdominal obesity has been proposed. However, the WC measurement alone could be an unsatisfactory marker because people with the same WC, but different height are unlikely to have the same risk [18]. A study performed among Spanish children explained the necessity to include the measure of WC in the clinical practice since the use of other anthropometric parameters underestimated the prevalence of abdominal obesity [19]. 

It has recently proposed that waist-to-height ratio (WtHR) may be a better indicator to identify risk owing to the fact that it is more sensitive as an early warning of health risk and allows the same boundary values among sex, age, and different ethnic groups [20,21,22]. Some studies have suggested that WtHR ≥ 0.5 is a valid predictor of higher cardiometabolic risk [23,24]; based on the idea that WC must be less than half your height [25]. This parameter has been proposed as an effective and simple measure of central adiposity. However, some studies have proposed different cutoff values of WtHR to detect with accuracy children at cardiometabolic risk [26,27], therefore, there is no worldwide consensus about the optimal cutoff of WtHR that should be used to identify children at risk. In addition, it has been proposed that it is advisable to use an alternative cutoff depending on the observed prevalence [28].

We aimed to determine the magnitude of association between WC and WtHR with cardiometabolic risk factors and MetS and WtHR cutoff associated with a more favorable cardiometabolic risk profile in Spanish children. 

## 2. Methods

### 2.1. Design and Study Participants

Data were taken from a cross-sectional survey conducted in 2010 among schoolchildren aged 8–11 years from 20 public schools in the province of Cuenca (Spain). The study methods have been reported elsewhere [29,30]. The final sample included 848 participants (51.9% girls). Sample size calculation indicated that the 848 schoolchildren included in the study were sufficient to detect a coefficient correlation of 0.20 between WtHR and MetS in a two-sided test (alpha ≤ 0.05, 95% power). This coefficient correlation is similar to the results reported from NHANES (National Health and Nutrition Examination Survey) data [8]. The Clinical Research Ethics Committee of the Virgen de la Luz Hospital in Cuenca approved the study protocol. After the study had been approved by the director and the board of governors of each school, a letter was sent to the parents to invite them to a meeting held to outline the study objectives. This letter also asked for the parents’ written consent to allow their children to participate in this study; and children gave verbal consent when, in informative talks held class by class, they were asked to collaborate. 

### 2.2. Measurements

Anthropometric variables and blood pressure measurements were recorded at schools by trained and certificated nurses. 

#### 2.2.1. Anthropometric Variables

Height and weight were measured by standard procedures. BMI was estimated as weight in kilograms divided by the square of the height in meters (kg/m^2^). WC was measured as the narrowest point between the lower costal border and the iliac crest using a metal tape measure, during shallow apnea with the children standing erect with abdomen relaxed in accordance with the guidelines of the International Society for the Advancement of Kinanthropometry [31]. WtHR was calculated as (WC (cm)/height (cm)).

#### 2.2.2. Blood Pressure

Systolic (SBP) and diastolic blood pressure (DBP) were determined by the average of two measurements taken within a 5 minutes interval after participants had rested for at least 5 minutes before the first measurement was taken. Participants were seated in a quiet, calm place with their right arm in a semi-flexed position at the heart level, using pediatric cuffs matched to the size of the child’s arm. Blood pressure was taken using an automated procedure and an OMRON M5-I. Categorization of blood pressure was done using gender-, age-, and height-specific cut points by using The Fourth Report on the Diagnosis, Evaluation, and Treatment of High Blood Pressure in Children and Adolescents [32]. Residual from these models represented height-adjusted SBP (_adj_SBP) and DBP (_adj_DBP). High blood pressure (stage 1) was defined as an SBP or DBP at the 95th percentile or higher but lower than the 99th percentile, high blood pressure (stage 2) was defined as an SBP or DBP at the 99th percentile or higher, and borderline blood pressure was defined as an SBP or DBP at the 90th percentile or higher but lower than the 95th percentile. To obtain more accurate results we simplified the four criteria obtained into two categories, as follows: normotensive (Percentile < 95th) and hypertensive (Percentile > 95th).

#### 2.2.3. Biochemical Assessments 

Blood samples were taken by puncturing the cubital vein under standard conditions between 8.15 and 9.00 a.m. after participants had fasted for at least 12 hours beforehand. Blood samples, which would take more than 75 minutes to reach the laboratory, were centrifuged in situ and transported refrigerated. Total cholesterol (TC), high-density lipoprotein cholesterol (HDL-C), low-density lipoprotein cholesterol (LDL-C), and triglycerides (TG) were determined. Adverse lipid concentrations were defined as follows: HDL-C concentrations <40 mg/dL (0.998 mmol/L) and TG concentrations >130 mg/dL (3.37 mmol/L) [33]. Fasting insulin and fasting glucose were determined using standard protocols. Insulin resistance was defined as insulin >15.05 µU/mL [34].

#### 2.2.4. Metabolic Syndrome (MetS) 

TG, HDL-C (inverted, iHDL-C), blood pressure (BP), and insulin were used to derive a composite cardiometabolic risk score as follows: Composite z-score = zTG + ziHDL-C + (z_adj_SBP + z_adj_DBP ^x^2)/3 + zInsulin. Each variable was converted into an age-adjusted z-score by gender. Cardiometabolic risk was defined as MetS ≥ 1SD [8,35].

### 2.3. Statistical Analyses

The continuous variables were expressed as the mean (standard) deviation and the categorical data as a frequency distribution. No parametric variables were transformed logarithmically for the analysis. Normality was verified using the Q–Q plots and histograms of each variable. Due to their skewed distribution, the following variables were log-transformed prior to analyses: insulin and TG. To aid interpretation, data were back-transformed from the log scale for presentation in the results. Participants were included in analysis if they had valid data for all outcome variables. For difference by gender. a Student’s t test was used for independent samples. We analyzed the differences in proportion distribution of each cardiometabolic risk category by sex using the software EPIDAT 4.2 (Regional Government of Galicia, Spain).

To describe the relationship between WtHR, WC, and cardiometabolic risk factors and MetS, the regression general lineal model was used, where the β coefficient is a marker of the change of the dependent variable and the partial ŋ^2^ is a marker of the proportion of overall variance of the dependent variable explained by the independent variable. Since multiple comparisons are made, in order to avoid incorrectly rejecting the null hypothesis, we applied the Bonferroni correction. This takes α = 0.05/K, where K is the number of predictors. Hence, with K = 5, the result of a comparison is considered significant if *p* < 0.01.

The receiver operating characteristic (ROC) curve was used to identify the best WtHR and WC cutoff. The predicted probabilities of each model were used in ROC analyses to determine each model’s discriminative capability. Sensitivity and specificity were calculated for a range of cutoffs for the measured WC and WtHR. The AUC for the ROC curves and their standard deviation and 95% confident intervals were calculated to determine the overall precision of anthropometric variables in diagnosing true positive participants and the values were compared [36]. Areas under the ROC curve (AUC) of 0.7 or smaller were considered to indicate low accuracy, thus a poor clinical utility. The decision for the optimal threshold for WtHR was the cutoff value with the highest accuracy that maximized the sum of the sensitivity and specificity [37]. For WC, the cutoff was calculated as the 90th percentile. Furthermore, the number of true positive (TP), true negative (TN), false positive (FP), false negative (FN), positive and negative predictive values (PPV and NPV) were also calculated. The diagnostic odds ratio (dOR) was calculated as a measure of the effectiveness of a diagnostic test. It is defined as the ratio of the odds of the test being positive if the subject has a disease relative to the odds of the test being positive if the subject does not have the disease [38].

Significance was set at *p* < 0.05 for all test. Analyses were performed using the statistical software package IBM SPSS Statistics 22.0 (SPSS, Inc., Chicago, IL, USA) and MedCalc statistical software (MedCalc Software Ltd, Mariakerke, Belgium).

## 3. Results 

A total of 848 children (440 girls, 51.9%) aged 8–11 years from 20 public schools of the province of Cuenca, were considered for data analysis. 

Clinical and cardiometabolic characteristics of sample according to sex are depicted in Table 1. Significant differences are observed for fasting glucose, insulin, TG, HDL-C, and SBP among girls and boys (*p* < 0.001).

Table 2 shows the variation of cardiometabolic risk factors and MetS according to the WtHR and WC, adjusted for sex and age. It was observed that WtHR explains about 14.4% of HDL-C variance, 17.8% of TG variance, 25.4% of insulin variance, 6.8% of SBP variance, 6.5% of DBP variance, and 34.4% of MetS variance (all values *p* < 0.001). On the other hand, we observed that WC explains about 15.2% of HDL-C variance, 16.9% of TG variance, 30.9% of insulin variance, 11.2% of SBP variance, 8.7% DBP variance, and 36.7% of MetS variance (all values *p* < 0.001).

**Figure 1:**
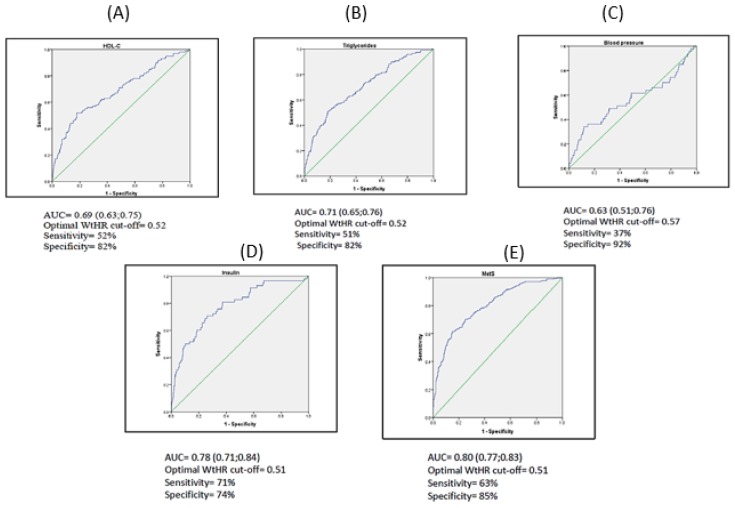
Representation AUC of WtHR and cardiometabolic parameters. (**A**) HDL-C; (**B**) Triglycerides; (**C**) Blood Pressure; (**D**) Insulin; (**E**) MetS.

**Figure 2:**
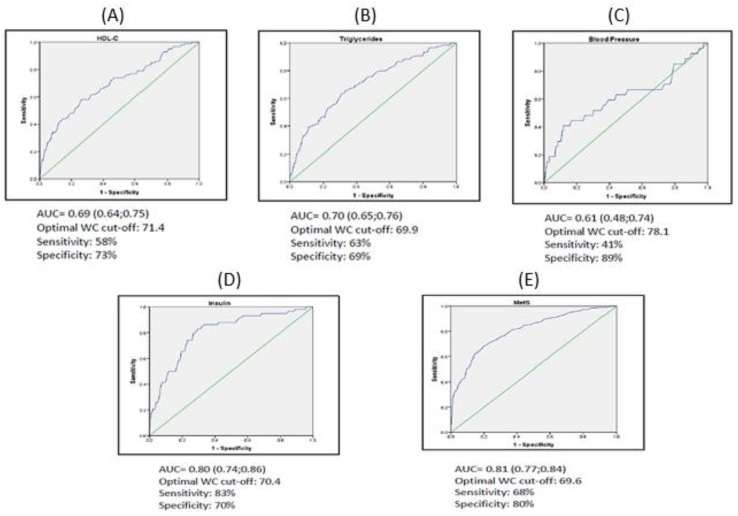
Representation AUC of WC and cardiometabolic parameters. (**A**) HDL-C; (**B**) Triglycerides; (**C**) Blood Pressure; (**D**) Insulin; (**E**) MetS.

Table 3 shows the associations between anthropometric variables with cardiometabolic risk factors (Figure 1 and Figure 2). When the analysis was conducted stratifying by sex equivalent results were found and no statistically significant differences were reported. The AUC with significant association ranged from 0.63 to 0.80. On the one hand, a WtHR higher than 0.52 was the optimal cutoff with 52% sensitivity and 82% specificity to classify children with low levels of HDL-C and a WtHR higher than 0.57 with 37% sensitivity and 92% specificity to classify children with high blood pressure. Both have a poor accuracy (AUC < 0.70) and have no clinical utility. A WtHR higher than 0.52 with 54% sensitivity and 79% specificity has clinical utility to classify children with high TG levels, 71% sensitivity and 74% specificity for a WtHR higher than 0.51 to classify children with high insulin levels, and a WtHR higher than 0.51 with 63% sensitivity and 85% specificity to classify children with MetS.

On the other hand, a WC higher than 80.2 cm presented high specificity, ranged from 91% to 99%, to classify children properly for all measured variables. However, it was observed that the measure of WC had a poor accuracy (AUC < 0.70) for HDL-C and blood pressure, therefore, it has no clinical utility. There were no significant differences for AUC between WtHR and WC for each cardiometabolic risk factor. By sex, there were also no significant differences. 

Table 4 shows the number of TP, TN, FP, and FN for different cutoff proposals. The sensitivity, specificity, PPV, and NPV for all measurements is also shown. The global accuracy for the optimal cut-off for WtHR ranged from 78% to 90%, whereas for WtHR higher than 0.5 the accuracy ranged from 67% to 75%. For WC, the global accuracy ranged from 76% to 89%. 

On the other hand, the dOR calculated for the optimal cutoff of WtHR varied from 4.28 to 9.07, for a WtHR higher than 0.5, the dOR varied from 2.95 to 7.36 and for WC, it varied from 5.30 to 27.40. The dOR is a measure of effectiveness of a diagnostic tool.

## 4. Discussion

The main result of the present study suggests that WtHR and WC show a similar association with cardiometabolic risk factors, especially for TG, insulin, and MetS (AUC > 0.70), to identify children with cardiometabolic abnormalities. 

Evidence indicates that dyslipidemia is a determinant factor for the appearance of atherosclerosis in the pediatric population [39]. The Healthy Study found the optimal cutoff of WtHR for HDL-C (69.2% sensitivity and 68.7% specificity) and TG (72.1% sensitivity and 68.9% specificity) was 0.52 for both variables [22] to identify children with abnormalities in lipid metabolism. A Brazilian study proposed a value of 0.48 as optimal WtHR cut-off for TG (65.8% sensitivity and 55% specificity) and 0.50 for HDL-C (60.0% sensitivity and 45.1% specificity) [40]. An Argentine study performed among children has proposed as optimal cutoff for TG as 0.50 (73% sensitivity and 72% specificity) and 0.48 for HDL-C (62% sensitivity and 62% specificity) [41]. However, a study performed in Brazil did not find an optimal WtHR cutoff to predict dyslipidemia among children aged 8–9 years [42]. Our results showed that the optimal cutoff for our population was 0.52 for TG (52% of sensitivity and 82% of specificity) and HDL-C (51% sensitivity and 82% specificity). We found lower sensitivities than the previous studies, but we obtained higher specificities. It is especially important to identify children with lipid abnormalities due to atherosclerosis processes. However, we did not find a statistically significant difference in diagnostic accuracy between WtHR and WC.

The global accuracy of the optimal WtHR cutoff (0.52) for HDL-C and TG is 78%, whereas that of the proposed WtHR (0.5) is 67% for HDL-C and 68% for TG, so that, the cutoff calculated presents a higher rate of true positive results than the proposed cutoff. The same occurs with diagnostic odds ratio, a higher odds ratio is observed for WtHR higher 0.52 than 0.50 for HDL-C and TG. However, the highest global accuracy and diagnostic odds ratio is observed for 90th percentile of WC. This is because the cutoff for WC presents a higher specificity than that for WtHR. 

The worldwide trend in BP levels is not clear. Several studies have measured the relation between BP and WtHR, showing mixed results. A study in China demonstrated that there is significant difference in BP among children and adolescents with different WtHR [43]. The Healthy Study [21] identified the optimal WtHR cutoff point among children as 0.54 with sensitivity of 40.5% and specificity as 71.7% to identify children with elevated BP. Our finding showed the optimal cutoff point was 0.57, we observed a value of sensitivity (37%) like the Healthy Study but we observed higher specificity (92%). 

To properly classify children with elevated BP, we can use a WtHR higher than 0.57 which presents a better global accuracy (90%) than the WtHR > 0.5 (67%) and WC (89%). The diagnostic odds ratio is two times greater than that observed for the proposed cutoff (WtHR > 0.5) and it also is higher than that for WC. However, our finding together with above-mentioned studies indicates that WtHR should not be used to classify children with elevated BP due to AUC values (<0.70). The WC should not be used either, due to the lack of clinical association (AUC < 0.70).

Insulin resistance is related to obesity and seems to have a significant role in the pathogenesis of MetS [44]. A Brazilian study [40] performed among children aged 6 and 10 years showed a value of 0.47 (92.6% sensitivity and 76.3% specificity) as optimal WtHR cutoff to identify children at risk. Our outcomes show that the optimal cutoff point to measure insulin resistance was 0.51 (71% sensitivity and 74% specificity) with an AUC of 0.78. No statistical difference was found for diagnostic accuracy between WtHR and WC. On the other hand, the global accuracy and diagnostic odds ratio were greater for the calculated cutoff (WtHR > 0.51) than for the proposed cutoff (WtHR > 0.5). Due to the lack of consensus to establish a cutoff point to define insulin resistance status, it is difficult to compare our findings with other studies. 

Based on the idea “keep your WC to less than half your height,” the cutoff of 0.5 was established [20]. However, different studies have demonstrated that this cutoff point has a low accuracy to diagnose metabolic abnormalities [28]. Based on the data from the NHANES, the cutoff of 0.60 has been proposed as an effective tool for classifying the metabolic risk among obese children and adolescents [26]. Another study performed among African adolescents proposed that the optimal cutoff point to indicate MetS was 0.41 [27]. For our population, we observed that the best cutoff point was 0.51 (63% sensitivity and 85% specificity) to identify children with cardiometabolic abnormalities. No statistical difference was found between WtHR and WC, and both were similar in discriminating children at risk. However, the diagnostic odds ratio was quite higher for WC than that for the WtHR, whereas WtHR > 0.51 presents better odds ratio than WtHR > 0.5 for diagnostic children at risk. The global accuracy was similar among different anthropometric variables.

The use of percentile 90th as a threshold of WC has been proposed for defining abdominal obesity. However, the WC measurement alone could be an unsatisfactory marker because people with the same WC but different height are unlikely to have the same risk [18]. A study performed among Spanish children explained the necessity to include the measure of WC in the clinical practice due to the fact that the use of other anthropometric parameters underestimated the prevalence of abdominal obesity [18]. We determinate the percentile 90th and the optimal cutoff based on the Youden index for WC, for each cardiometabolic risk factors and MetS score in our population and the threshold values of p90 were higher than those calculated using the Youden index. Moreover, the sensitivity, which can be defined as the capacity of a test to properly classify unhealthy persons, for this threshold value was lower than it was based on the Youden index. However, we noticed a higher specificity, so that the percentile 90th value could be used as a confirmatory tool instead of as a screening tool. We also calculated the dOR as a single indicator of test performance. It is not prevalence dependent and may be easier to understand, as it is a familiar epidemiologic measure [38].

The WtHR has been proposed as a good anthropometric parameter for screening because of its association with cardiometabolic risk factors [25]. Different studies have proposed that WtHR is more sensitive than other anthropometric parameters as an early warning of health risks [11] and seems to be an effective indicator of central adiposity [26]. Children with a high level of WtHR have four times more risk of developing MetS [28]. Moreover, it is simple to measure and calculated because it does not require gender- and age-specific centiles, but the threshold of WtHR ≥ 0.5 has not yet been established as the optimal cutoff for all populations and ethnicities [27]. Our results show WC and WtHR were similar in discriminating children at risk as has already been described in the literature [45]. However, we observed the capacity of a WtHR cutoff ≥ 0.5 to properly classify children at risk is lower than that of the optimal calculated cutoff for our population. 

This study has some limitations. The main limitation of this study is the lack of pubertal stage among the covariates; although it was measured, the lack of variability did not let us use it as a covariate. Secondly, it is a cross-sectional survey, which does not permit the establishment of a cause–effect relationship. Thirdly, we did not have access to data on other factors that could influence our outcomes such as familiar history. Fourthly, for WC measurement, we did not control the posture, phase of respiration, and the time since the last meal. Insulin is an alternative to measure insulin resistance and the lack of consensus of optimal cutoff makes the comparison among studies difficult. Associating independently against each variable can lead to spurious conclusions and can inflate Type I error. Finally, we only analyzed children aged 8–11 from Cuenca; extrapolation of the results to other age groups and geographic settings is not robust and should be done with caution. Despite the constraints, it is of note that our analyses are based on data that are comparable over time in terms of population representativeness and measurement techniques and are unique in Spain for examining WtHR cutoffs among children.

## 5. Conclusions

In summary, WtHR and WC could be used as non-invasive approaches to identify children with metabolic abnormalities according to the international cutoff, due to low cost, simplicity, and non-invasive procedures; moreover, it is an accurate marker of central obesity. Our study also suggests that WC did not provide superior identification of high cardiometabolic risk as compared with WtHR. In addition, the use of WtHR as a tool to detect the population at risk offers some advantages as an early risk measure and effective central adiposity tool, by not requiring reference charts, and being less influenced by sexual maturity. The sensitivity values of cutoffs points observed has a clinical utility, because it could be used as a screening tool in public schools to identify those children who should be examined with direct measurements. However, it needs to be considered that the low sensitivity values could misclassify a sizeable proportion of children with increased cardiometabolic risk. Therefore, longitudinal studies are necessary to determine whether reducing WtHR and WC decreases the likelihood of MetS and cardiometabolic risk factors.

## Figures and Tables

**Table 1:** Characteristics of the study sample, by gender.

	Total (*n* = 848)	Boys (*n* = 408)	Girls (*n* = 440)	*p*
Age	9.5 ± 0.7	9.5 ± 0.7	9.5 ± 0.7	0.986
Anthropometric measures				
Weight (kg)	37.3 ± 9.1	37.6 ± 9.3	37.0 ± 8.9	0.331
Height (cm)	139.6 ± 7.0	139.5 ± 6.8	139.7 ± 7.2	0.713
BMI (kg/m^2^)	19.0 ± 3.6	19.1 ± 3.7	18.8 ± 3.6	0.183
WC (cm)	67.5 ± 9.2	68.2 ± 9.5	66.8 ± 8.8	0.025
WtHR	0.5 ± 0.06	0.5 ± 0.06	0.5 ± 0.06	0.011
Biochemical measurements				
Glucose (mg/dL)	83.1 ± 6.3	84.3 ± 6.3	82.0 ± 6.1	<0.001
Insulin (µU/mL) *	6.9 (5.1, 9.4)	6.3 (4.8, 8.5)	7.5 (5.6, 10.2)	<0.001
TG (mg/dL) *	58 (44, 81)	55 (41, 77)	62 (47.3, 84)	<0.001
HDL-C (mg/dL)	59.9 ± 13.4	61.6 ± 14.0	58.3 ± 12.7	<0.001
SBP (mmHg)	100.6 ± 9.0	101.9 ± 9.1	99.4 ± 8.8	<0.001
DBP (mmHg)	62.0 ± 7.3	61.9 ± 7.3	62.1 ± 7.2	0.767
MetS	0.01 ± 2.4	0.04 ± 2.4	−0.02 ± 2.4	0.692
Frequency of metabolic abnormalities	% (*n*)			
HDL-C (<40mg/dL)	11.8 (100)	9.1 (37)	14.3 (63)	0.018
TG (>130mg/dL)	14 (119)	10.8 (44)	17 (75)	0.009
Elevated BP (P> 95)	3.2 (27)	3.7 (15)	2.7 (12)	0.432
Insulin (>15.05 µU/mL)	6.8 (58)	4.9 (20)	8.6 (38)	0.030
MetS (>+1SD)	33.3 (282)	35.5 (145)	31.2 (137)	0.174

Data are presented as mean ± standard deviation (95% confident intervals); * Data are presented as median (interquartile range: 25th–75th percentile); BMI = body mass index; WC = waist circumference; WtHR= waist-to-height ratio; TG = triglycerides; HDL-C = high-density lipoprotein cholesterol; SBP = systolic blood pressure; DBP = diastolic blood pressure; BP = blood pressure P = percentile; MetS = metabolic syndrome index.

**Table 2:** Variation of metabolic parameters according to weight-to-height ratio (WtHR) and waist circumference (WC), adjusted for sex and age.

	Independent Variable: WtHR			Independent Variable: WC		
Dependent Variable	B (SE)	Partial ŋ^2^	*p*	B (SE)	Partial ŋ^2^	*p*
HDL-C (mg/dL)	−0.86 (0.07)	0.144	<0.001	−0.57(0.05)	0.152	<0.001
TG (mg/dL)	0.01 (0.001)	0.178	<0.001	0.01 (0.001)	0.169	<0.001
Insulin (µU/mL)	0.20 (0.001)	0.258	<0.001	0.01(0.001)	0.309	<0.001
SBP (mmHg)	0.39 (0.05)	0.068	<0.001	0.33 (0.03)	0.112	<0.001
DBP (mmHg)	0.32 (0.04)	0.065	<0.001	0.23 (0.03)	0.087	<0.001
MetS	0.24 (0.01)	0.344	<0.001	0.16 (0.01)	0.367	<0.001

B, coefficients non-standardized (the change of the dependent variable for a unitary increase of the independent variable); SE: standard error; Partial-Eta^2^, portion of overall variance of the dependent variable explained by the independent variable.; TG and insulin were log transformed.

**Table 3:** Comparison between area under the receiver operating characteristic (ROC) curve of WtHR calculated and WC, and standardized WtHR and WC, adjusted for, age as indicators of metabolic abnormalities in children.

WtHR and WC calculated											
	AUC	95% CI	WtHR	Sens.	Spec.	AUC	95% CI	WC (P90)	Sens.	Spec.	AUC Difference (SE)
HDL-C	0.69	(0.63; 0.75)	0.52	52	82	0.69	(0.64; 0.75)	80.2	30	93	0.01 (0.01)
TG	0.71	(0.65;0.76)	0.52	51	82	0.70	(0.65; 0.76)	80.2	28	93	0.01 (0.01)
BP	0.63	(0.51;0.76)	0.57	37	92	0.61	(0.48; 0.74)	80.2	30	91	0.02 (0.02)
Insulin	0.78	(0.71;0.84)	0.51	71	74	0.80	(0.74; 0.86)	80.2	41	92	0.02 (0.01)
MetS	0.80	(0.77;0.83)	0.51	63	85	0.81	(0.77; 0.84)	80.2	28	99	0.01 (0.01)
HDL-C	0.69	(0.63;0.75)	0.50	57	69	0.69	(0.64; 0.75)	80.2	30	93	0.01 (0.01)
TG	0.71	(0.65;0.76)	0.50	58	70	0.70	(0.65; 0.76)	80.2	28	93	0.01 (0.01)
BP	0.63	(0.51;0.76)	0.50	56	67	0.61	(0.48; 0.74)	80.2	30	91	0.02 (0.02)
Insulin	0.78	(0.71;0.84)	0.50	72	69	0.80	(0.74; 0.86)	80.2	41	92	0.02 (0.01)
MetS	0.80	(0.77;0.83)	0.50	64	81	0.81	(0.77; 0.84)	80.2	28	99	0.01 (0.01)

AUC: area under the ROC; Sens: sensitivity; Spec: specificity; SE: standard error.

**Table 4:** Statistical parameters associated with different proposed cut-offs for each anthropometric measure.

	WtHR	TP	TN	FP	FN	Sens.	Spec.	PPV	NPV	Accuracy	dOR	95% CI
HDL-C	0.52	52	597	151	48	52	82	26	93	78	4.28	(2.78; 6.59)
TG	0.52	61	587	142	58	51	82	30	91	78	4.35	(2.90; 6.51)
BP	0.57	9	754	67	18	37	92	12	98	90	5.63	(2.43; 13.01)
Insulin	0.51	40	588	202	18	71	74	17	97	74	6.47	(3.63; 11.54)
MetS	0.51	164	492	78	114	63	85	68	81	78	9.07	(6.47; 12.72)
HDL-C	0.5	57	516	232	43	57	69	20	92	67	2.95	(1.93; 4.51)
TG	0.5	69	509	220	50	58	70	24	91	68	3.19	(2.15; 4.75)
BP	0.5	15	547	274	12	56	67	1	98	67	2.50	(1.15; 5.41)
Insulin	0.5	42	543	247	16	72	69	15	97	69	5.77	(3.18; 10.46)
MetS	0.5	178	459	111	110	64	81	62	82	75	7.36	(5.34; 10.15)
HDL-C	80.2	30	692	56	70	30	93	35	91	85	5.30	(3.19-8.79)
TG	80.2	33	676	53	86	28	93	38	89	84	4.89	(3.00; 7.98)
BP	80.2	8	743	78	19	30	91	9	98	89	4.01	(1.7; 9.40)
Insulin	80.2	24	728	62	34	41	92	28	96	88	8.29	(4.63; 14.85)
MetS	80.2	78	562	8	200	28	99	91	74	76	27.40	(13.00; 57.73)

WC = waist circumference; WtHR = waist-to-height ratio; TG = triglycerides; HDL-C = high-density lipoprotein cholesterol; BP = blood pressure; MetS = metabolic syndrome index. The abbreviations TP, FP, FN, and TN denote the number of respectively, true positives, false positives, false negatives, and true negatives. PPV = positive predictive value; NPV = negative predictive value; Sens = sensitivity; Spec = specificity; dOR = diagnostic odds ratio.
